# Prenatal metal mixtures and child blood pressure in the Rhea mother-child cohort in Greece

**DOI:** 10.1186/s12940-020-00685-9

**Published:** 2021-01-06

**Authors:** Caitlin G. Howe, Katerina Margetaki, Marina Vafeiadi, Theano Roumeliotaki, Marianna Karachaliou, Manolis Kogevinas, Rob McConnell, Sandrah P. Eckel, David V. Conti, Maria Kippler, Shohreh F. Farzan, Leda Chatzi

**Affiliations:** 1 Department of Epidemiology, Geisel School of Medicine at Dartmouth, Dartmouth College, 1 Medical Center Dr, Lebanon, NH 03766 USA; 2 Department of Preventive Medicine, University of Southern California, Los Angeles, CA USA; 3 Department of Social Medicine, Faculty of Medicine, University of Crete, Heraklion, Crete Greece; 4 ISGlobal, Barcelona, Spain; 5 Universitat Pompeu Fabra, Barcelona, Spain; 6 CIBER Epidemiología y Salud Pública, Madrid, Spain; 7 Institute of Environmental Medicine, Karolinska Institutet, Stockholm, Sweden

**Keywords:** Metals, Mixtures, Blood pressure, Prenatal, Childhood

## Abstract

**Background:**

Child blood pressure (BP) is predictive of future cardiovascular risk. Prenatal exposure to metals has been associated with higher BP in childhood, but most studies have evaluated elements individually and measured BP at a single time point. We investigated impacts of prenatal metal mixture exposures on longitudinal changes in BP during childhood and elevated BP at 11 years of age.

**Methods:**

The current study included 176 mother-child pairs from the Rhea Study in Heraklion, Greece and focused on eight elements (antimony, arsenic, cadmium, cobalt, lead, magnesium, molybdenum, selenium) measured in maternal urine samples collected during pregnancy (median gestational age at collection: 12 weeks). BP was measured at approximately 4, 6, and 11 years of age. Covariate-adjusted Bayesian Varying Coefficient Kernel Machine Regression and Bayesian Kernel Machine Regression (BKMR) were used to evaluate metal mixture impacts on baseline and longitudinal changes in BP (from ages 4 to 11) and the development of elevated BP at age 11, respectively. BKMR results were compared using static versus percentile-based cutoffs to define elevated BP.

**Results:**

Molybdenum and lead were the mixture components most consistently associated with BP. J-shaped relationships were observed between molybdenum and both systolic and diastolic BP at age 4. Similar associations were identified for both molybdenum and lead in relation to elevated BP at age 11. For molybdenum concentrations above the inflection points (~ 40–80 μg/L), positive associations with BP at age 4 were stronger at high levels of lead. Lead was positively associated with BP measures at age 4, but only at high levels of molybdenum. Potential interactions between molybdenum and lead were also identified for BP at age 11, but were sensitive to the cutoffs used to define elevated BP.

**Conclusions:**

Prenatal exposure to high levels of molybdenum and lead, particularly in combination, may contribute to higher BP at age 4. These early effects appear to persist throughout childhood, contributing to elevated BP in adolescence. Future studies are needed to identify the major sources of molybdenum and lead in this population.

**Supplementary Information:**

The online version contains supplementary material available at 10.1186/s12940-020-00685-9.

## Background

Elevated blood pressure (BP) is an established risk factor for cardiovascular disease (CVD) [1]. Longitudinal studies following children into adulthood have observed that even in early life, BP is predictive of future cardiovascular risk [2]. For example, elevated BP in childhood has been associated with increased risk for hypertension and premature death from coronary heart disease, as well as intermediate outcomes, including left ventricular hypertrophy and increased carotid intima-media thickness [2–4]. Higher BP levels in childhood have also been associated with lower cognitive test scores in both early adulthood and mid-life [5]. It is therefore critical to identify modifiable factors that influence BP in early life.

There is substantial evidence supporting a link between exposure to metals, metalloids, and metalloid-like elements (hereafter collectively referred to as “metals”) and risk for hypertension and CVD [6–9]. In fact, a representative study of the general population in the United States has estimated that molybdenum (Mo), lead (Pb), and antimony (Sb) each contribute to ~ 6–7% of the population attributable risk for high BP [8]. Several studies have also reported that arsenic (As) and cadmium (Cd) exposures increase risk for elevated BP and hypertension in adult populations [10–12]. Essential elements, including cobalt (Co), magnesium (Mg), and selenium (Se), have also been associated with BP levels in adults [13–18]. However, these relationships may be complex, as these elements have critical physiological functions, but can be toxic at high levels [19, 20].

Although more limited, a growing body of evidence suggests that metal exposures also contribute to elevated BP in children and adolescents [21–25]. Exposure to toxic metals during the prenatal period may be particularly detrimental, as fetal development consists of a series of carefully-timed events, and dysregulation of these processes can have long-lasting consequences [26]. In support of this, several studies have reported positive associations between toxic metal exposures during pregnancy and BP levels in childhood [27–30]. However, most of these studies evaluated metals individually (focusing on Pb) and measured BP at a single time point. Far less is known about the impacts of complex metal mixtures, which are more representative of human exposures, on longitudinal changes in BP during childhood.

In the current study, we focused on a cohort of children in Greece [31], a country with a high prevalence of pediatric hypertension [32–35]. We examined the impact of prenatal exposure to a complex mixture of metals on 1) longitudinal changes in BP across 7 years of follow-up (ages 4 to 11) and 2) risk of elevated BP at 11 years of age. We investigated eight metals (As, Cd, Co, Mg, Mo, Pb, Sb, Se) that have been associated individually with BP in either children or adults and used flexible mixture modeling approaches that can capture complex non-linear relationships and possible synergistic and antagonistic relationships between mixture components [36, 37], including a novel approach that can accommodate longitudinal data [37].

## Methods

### Study participants

The current study focused on a subset of mother-child pairs from the Rhea Study, a longitudinal cohort in Heraklion, Crete, Greece [31]. Briefly, participants were included in Rhea if they were pregnant, residents of the study area, 16 years of age or older, and had no communication handicap. Participants were recruited in early pregnancy at the time of their first major ultrasound examination (< 15 weeks’ gestation). The current study focused on 176 mother-child pairs with urinary metal measurements (excluding extreme outliers based on the mean ± 4 SD), child BP measures at all three time points (4, 6, and 11 years of age), and complete covariate information (Fig. S1). This study was conducted according to the principles of the Declaration of Helsinki and was approved by the ethical committee of the University Hospital in Heraklion, Greece and the Regional Ethical Review Board in Stockholm, Sweden. Informed consent was obtained from all participants.

### Urine collection and urine metals analysis

In early pregnancy (median (IQR): 12 [11, 15] weeks’ gestation), maternal spot urine samples were collected in sterile, polypropylene urine cups. These urine samples were aliquoted into 4 ml cryotube vials (Thermo Fisher Scientific, USA) and stored at − 80 °C. A panel of 15 metals was measured in urine by inductively coupled plasma mass spectrometry (Agilent 7700X; Agilent Technologies, Tokyo, Japan) with an Octopole Reaction System at the Institute of Environmental Medicine, Karolinska Institutet, Stockholm, Sweden. Urine samples were diluted 1:10 in 1% nitric acid (prepared from 65% Suprapur, Merck, Darmstadt, Germany). For the current study, we focused on a subset of eight metals [As (*m/z* 75), Cd (*m/z* 111), Co (*m/z* 59), Mg (*m/z* 24), Mo (*m/z* 95), Pb (*m/z* 202), Sb (*m/z* 121), Se (*m/z* 75)] that have previously been associated individually with BP and for which urine is considered an acceptable biomarker of exposure [8, 11–16, 21–25, 27–30, 38–45]. The one exception to this second criterion was Pb, as blood is the preferred biomarker of exposure [46]. However, we retained Pb, because urine can capture inter-individual differences in exposure [46], and Pb has been associated with elevated BP in both children and adults across multiple populations [8, 27, 29, 30, 38]. The limits of detection (LOD) for As, Cd, Co, Mg, Mo, Pb, Sb, and Se were < 0.03 μg/L, < 0.001 μg/L, < 0.001 μg/L, < 1.2 μg/L, < 0.03 μg/L, < 0.003 μg/L, < 0.002 μg/L, and < 0.014 μg/L, respectively. One sample had a Sb concentration below the LOD; the machine value for Sb was retained for this sample. All other samples were above the LOD for each metal. Quality control was performed by including two commercial control materials (Seronorm™ Trace Elements Urine Blank, REF 201305, LOT OK4636 and Seronorm™ Trace Elements Urine, REF 201205, LOT NO2525) in each analytical run. Overall, the obtained urinary element concentrations showed good agreement with the reference value for each element (Table S1).

### Specific gravity

Urinary specific gravity was measured using a digital refractometer (EUROMEX RD712 Clinical Refractometer; Euromex Microscopen BV, Arhnem, Holland). To account for urine dilution, urinary metal concentrations were adjusted for specific gravity using the following formula: urinary concentration × [(mean specific gravity (1.020) − 1)/(individual specific gravity − 1)]) [47].

### Child BP measures

Trained research assistants measured systolic BP (SBP) and diastolic BP (DBP) at approximately 4, 6, and 11 years of age. After 5 min of rest in a seated position, BP measures were obtained using a Dinamap automated oscillometric recorder (Dinamap Pro Care 400, Critikon, Tampa, FL) from the child’s right arm with a cuff that was of appropriate size for the child’s arm circumference. A minimum of three BP measurements were obtained at each visit, taken one minute apart. The average of these measurements was calculated for both SBP and DBP [48]. For primary analyses, elevated BP at 11 years of age was defined as a SBP measure ≥110 mmHg and/or a DBP measure > 70 mmHg [49]. Xi et al. have previously proposed this definition for elevated BP for children aged 6–11, because these static cutoffs are easier to implement in clinical settings [49]. This definition of elevated BP in childhood is similarly predictive of hypertension and preclinical CVD in adulthood as the American Academy of Pediatrics (AAP) definition, which uses percentile-based cutoffs [49, 50]. In sensitivity analyses, we also examined elevated BP at age 11 defined using the AAP guidelines (i.e., SBP and/or DBP greater than or equal to the 90th percentile for sex, age, and height) [51].

### Covariate information

Personal interviews combined with self-administered questionnaires and medical records were used to obtain information on potential confounders and precision variables. These included maternal age at urine collection, duration of maternal education at recruitment (≤6 years, > 6 years and < 12 years, or ≥ 12 years), maternal pre-pregnancy BMI (kg/m^2^), and maternal ever tobacco smoke use during pregnancy (reported at 12 weeks’ gestation). Child characteristics included: age, sex, height, BMI, and environmental tobacco smoke (ETS) exposure in childhood at each time point (4, 6, 11 years), which was defined as any member of the household smoking more than one cigarette inside the home at the time of the interview. Child height and weight were measured by trained research assistants using a validated scale (Seca Bellisima 841 scale; Seca GmbH & Co. KG, Hamburg, Germany) according to standard operating procedures. Child overweight and obesity were estimated using the International Obesity Task Force guidelines [52]. In preliminary models, we also evaluated the impact of additionally adjusting models for the frequency (number of times per week) of maternal fish and seafood consumption during pregnancy for the 150 participants who had this information available. Maternal fish and seafood consumption was determined using a food frequency questionnaire administered at recruitment [53]. Results were similar after this additional adjustment, so this covariate was excluded from final models.

### Statistical analyses

Statistical analyses were conducted using Stata 16 and R (Version 3.6.2). Descriptive statistics were calculated for participant demographics, urinary metal concentrations, and BP measures. Because the urinary metals were largely right-skewed, they were log_2_-transformed to reduce the influence of extreme values. These measures were then mean-centered and scaled. Pearson correlations were used to evaluate relationships between each pair of metals. Because we hypothesized a priori that 1) essential elements (Co, Mg, Mo, Se) would have non-linear relationships with BP, 2) toxic and essential elements would act in opposing directions, and 3) some metals would act synergistically or antagonistically, we used two mixture modeling approaches that can accommodate and examine these scenarios. We used Bayesian Varying Coefficient Kernel Machine Regression (BVCKMR) to evaluate prenatal metal mixture impacts on DBP and SBP at age 4 (baseline) and also on longitudinal changes in these BP measures during childhood. BVCKMR is a recently developed approach that, unlike most environmental mixture methods, can accommodate longitudinal data by estimating associations between mixtures and health outcome trajectories [37]. To evaluate the impact of prenatal metal mixtures on elevated BP at 11 years of age, we used a similar mixture modeling method, Bayesian Kernel Machine Regression (BKMR) [36], which was primarily designed for evaluating outcomes measured at a single time point.

The BVCKMR model is defined as

$$ {y}_{\mathrm{i}j}={\gamma}_1+{\gamma}_2\times {\mathrm{age}}_{\mathrm{i}j}+{h}_1\left({\mathrm{z}}_{1\mathrm{i}},\cdots, {\mathrm{z}}_{\mathrm{Mi}}\right)+{h}_2\left({\mathrm{z}}_{1\mathrm{i}},\dots, {\mathrm{z}}_{\mathrm{Mi}}\right)\times {\mathrm{age}}_{\mathrm{i}j}+{\mathbf{x}}_{\mathbf{i}}^{\mathrm{T}}\boldsymbol{\beta} +{{\mathrm{u}}_{\mathrm{i}j}}^{\mathrm{T}}{\mathrm{b}}_{\mathrm{i}}+\in \mathrm{i}j $$

where the outcome y_ij_ is related to the exposure mixture z_i_ = (z_1i_,...,z_Mi_)^T^ through two flexible functions h_1_(·) and h_2_(·), controlling for potential confounders x_i_ = (x_1i_,...,x_pi_) [37]. The *h*_*1*_ function represents the association between the mixture and the BP measures at baseline (age 4) while *h*_*2*_ represents how the mixture modifies the annual rate of change in the BP measure over time (age 4 to 11). This BVCKMR model assesses the directionality and relative importance of each mixture component on linear health outcome trajectories, while accounting for possible nonlinear relationships between each exposure and outcome and non-additive effects of the mixture components [37]. The relative importance of each mixture component is defined as the difference in the outcome, comparing the individual metal of interest at high levels (75th percentile) versus low levels (25th percentile), while holding all other metals constant at their median values. Metal-outcome associations were considered statistically significant if the posterior credible interval for the effect estimate did not span 0. We ran 100,000 MCMC iterations, using the first half of iterations as burn-in.

The BKMR model is defined as

$$ {y}_{\mathrm{i}}=h\left({\mathrm{z}}_{1\mathrm{i}},\cdots, {\mathrm{z}}_{\mathrm{Mi}}\right)+{\mathbf{x}}_{\mathbf{i}}^{\mathrm{T}}\boldsymbol{\beta} +\in \mathrm{i} $$

where function *h*() represents the kernel exposure-response machine function, coefficients ***β***^*T*^ represent effect estimates for the *X*th covariate for the *i*th individual, and ε_*i*_ represents the model residuals [36]. Using the “bkmr” R package, we chose the hierarchical variable selection option, grouping elements into toxic (As, Cd, Pb, Sb) and essential (Co, Mg, Mo, Se) elements, given a priori hypotheses that metals within each group would similarly impact BP (i.e., adverse effects for toxic metals and non-linear relationships for essential elements), and ran 100,000 MCMC iterations. The first half of iterations was used as burn-in. To reduce potential autocorrelation, we thinned the chains, selecting every 10th iteration. Model convergence was visually inspected using trace plots. Posterior inclusion probabilities (PIPs) were used to rank the importance of each mixture component.

BKMR and BVCKMR models were adjusted for hypothesized confounders and precision variables, identified using directed acyclic graphs (DAGs) (Fig. S2). Final BKMR and BVCKMR models were adjusted for the minimum set of potential confounders necessary to close all backdoor paths between the exposure and outcome: maternal age (continuous), maternal education (categorical: ≤6 years, > 6 years and < 12 years, or ≥ 12 years), maternal pre-pregnancy BMI (continuous), and maternal smoking during pregnancy (binary: ever versus never). Models were also adjusted for three potential precision variables that were prioritized because they are known to be important predictors of BP in children [51]: child sex (binary: female versus male), child’s exact age (continuous), and child height (continuous) at the relevant time points (11-year time point only for BKMR; 4-, 6-, and 11-year time points for BVCKMR). Given the high prevalence of ETS exposure among children in Greece [54, 55], we also examined results from both BVCKMR and BKMR models after additionally adjusting for this potential precision variable in sensitivity analyses (ETS exposure at each time point for BVCKMR and ETS exposure at age 11 for BKMR). Child BMI was identified as a potential collider based on our DAG (Fig. S2), and was therefore excluded from all models. In sensitivity analyses, we compared BKMR results when using the AAP percentile-based cutoffs [51] with the static cutoffs proposed by Xi et al. [49] to define elevated BP at age 11.

For confirmatory analyses, we compared results from BVCKMR (for SBP and DBP measures) using generalized additive mixed models (GAMMs), in which each metal was evaluated individually. Similarly, BKMR results (for elevated BP at age 11) were compared with results from generalized additive models (GAMs). We conducted GAMMs and GAMs using the “mgcv” R package [56]. These models were adjusted for the same set of covariates as the BVCKMR and BKMR models, respectively. Interactions between pairs of metals were examined for both sets of models, and statistical significance was determined for the individual metal associations and pairwise interactions using a *p*-value threshold of 0.05.

## Results

**Fig. 1:**
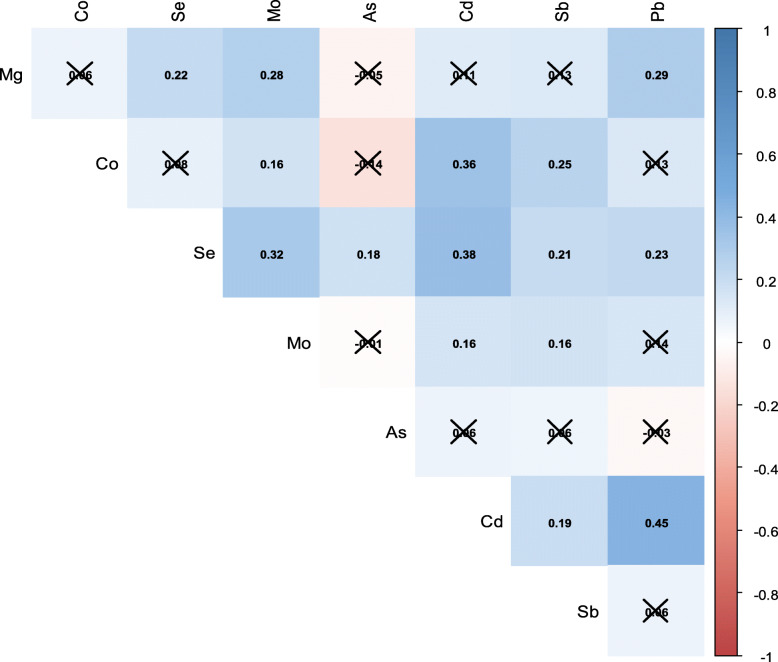
Pearson Correlation Coefficients for Urinary Metal Pairs. Metals were log_2_-transformed, mean-centered, and scaled. Blue shades indicate positive correlations and red shades indicate negative correlations, as indicated in the key. X indicates that the correlation was not statistically significant (*p*-value ≥0.05). Abbreviations used: As, arsenic; Cd, cadmium; Co, cobalt; Mg, magnesium; Mo, molybdenum; Pb, lead; Sb, antimony; Se, selenium

Participant characteristics are shown in Table 1. The mean (SD) maternal age and pre-pregnancy BMI was 30 (4) years and 24.0 (4.0) kg/m^2^, respectively, and 23% of the women reported ever smoking during the pregnancy. There were more female (55.7%) than male (44.3%) children in the study sample. The mean (SD) BMI at age 4, 6, and 11 was 16.4 (2.0) kg/m^2^,16.8 (2.7) kg/m^2^, and 20.4 (4.1) kg/m^2^, respectively. There was a high prevalence of overweight/obesity at each age: 35 (19.9%) at age 4, 49 (27.8%) at age 6, and 78 (44.3%) at age 11. On average, BP measures increased during childhood. The mean (SD) SBP was 90.6 (7.6) mmHg, 94.0 (8.2) mmHg, and 105.8 (9.4) mmHg at ages 4, 6, and 11, respectively. For the same ages, the mean (SD) DBP was 53.7 (5.0) mmHg, 53.9 (6.4) mmHg, and 61.2 (6.9) mmHg, respectively. The prevalence of elevated BP at the 11-year time point was 30.7% when using the static cutoffs proposed by Xi et al. [49] and 17.6% when using the percentile-based cutoffs proposed by the AAP [51]. Overall, the study sample was similar to the larger Rhea Cohort, although participating mothers were slightly older and more educated compared with women who did not meet the inclusion criteria (Table S2). There were also some very small, although statistically significant, differences between the two groups for DBP at age 6 and for SBP at age 11 (Table S2). Urinary metal concentrations for study participants are shown with and without adjustment for specific gravity in Table 2, and Pearson correlations between metal pairs are shown in Fig. 1. Most of the metals were positively correlated with each other, although correlations were generally weak to moderate, ranging from ±0.01 to ±0.45. The strongest correlation was observed between Cd and Pb (*r =* 0.45, *p* < 0.01).

**Table 1:** Characteristics of 176 Mother-Child Pairs from the Rhea Cohort

Participant Characteristic	N (%) or Mean (SD)		
**Maternal Characteristics**			
Maternal Age, years		30.3 (4.2)	
Pre-Pregnancy BMI, kg/m^2^		24.0 (4.0)	
Maternal Education			
Low (≤6 years)		18 (10.2)	
Medium (> 6 years and < 12 years)		90 (51.1)	
High (≥12 years)		68 (38.6)	
Smoking During Pregnancy			
Non-Smoker		136 (77.3)	
Smoker		40 (22.7)	
Frequency of Fish and Seafood Consumption^a^, Times/Week		1.0 (0.6)	
**Child Characteristics**			
Sex			
Male		98 (55.7)	
Female		78 (44.3)	
Elevated Blood Pressure at Age 11 (Static Cutoffs)^b^			
Elevated		54 (30.7)	
Normal		122 (69.3)	
Elevated Blood Pressure at Age 11 (Percentile Cutoffs)^c^			
Elevated		31 (17.6)	
Normal		145 (82.4)	
**Repeated Measurements**	** 4 Years**	**6 Years**	**11 Years**
Exact Age, years	4.2 (0.2)	6.5 (0.3)	10.9 (0.3)
Height, cm	104.5 (4.2)	120.0 (4.8)	144.3 (6.3)
BMI, kg/m^2^	16.4 (2.0)	16.8 (2.7)	20.4 (4.1)
Overweight	25 (14.2)	35 (19.9)	58 (33.0)
Obese	10 (5.7)	14 (8.0)	20 (11.4)
Environmental Tobacco Smoke Exposure	74 (42.8)	64 (36.4)	45 (25.6)
Systolic Blood Pressure, mmHg	90.6 (7.6)	94.0 (8.2)	105.8 (9.4)
Diastolic Blood Pressure, mmHg	53.7 (5.0)	53.9 (6.4)	61.2 (6.9)

^a^*n* = 150

^b^Elevated blood pressure at age 11 was defined using the static cutoffs recommended by Xi et al. [49]: systolic blood pressure ≥ 110 mmHg and/or a diastolic blood pressure > 70 mmHg

^c^Elevated blood pressure was defined as systolic blood pressure or diastolic blood pressure ≥ 90th percentile for sex, age, and height, according to the 2017 American Academy of Pediatrics guidelines [51]

**Table 2:** Early Pregnancy Maternal Urinary Metal Concentrations (*n* = 176)

Urinary Metal Concentrations	Geometric Mean (95%CI)	p10	p25	p50	p75	p90	n < LOD
**Unadjusted**							
Magnesium (mg/L)	59.6 (52.4, 67.7)	16.6	35.7	76.1	11.1	14.5	0
Cobalt (μg/L)	0.48 (0.41, 0.56)	0.11	0.26	0.44	1.07	1.97	0
Selenium (μg/L)	19.4 (17.3, 21.6)	6.0	13.0	22.8	33.8	41.8	0
Molybdenum (μg/L)	58.15 (51.90, 65.16)	20.9	35.8	61.0	98.4	145.8	0
Arsenic (μg/L)	13.55 (11.01, 16.68)	2.7	5.2	12.1	30.5	80.1	0
Cadmium (μg/L)	0.41 (0.35, 0.47)	0.11	0.23	0.44	0.77	1.21	0
Antimony (μg/L)	0.05 (0.04, 0.05)	0.02	0.03	0.05	0.08	0.11	1
Lead (μg/L)	0.82 (0.71, 0.96)	0.20	0.52	1.06	1.56	2.32	0
**SG-Adjusted**							
Magnesium (mg/L)	66.9 (61.3, 72.9)	30.9	50.5	71.3	100.9	133.4	0
Cobalt (μg/L)	0.54 (0.48, 0.61)	0.22	0.28	0.46	1.01	1.78	0
Selenium (μg/L)	21.72 (20.72, 22.77)	15.2	17.3	21.8	27.0	31.9	0
Molybdenum (μg/L)	65.26 (61.13, 69.66)	38.1	50.5	64.9	86.7	114.1	0
Arsenic (μg/L)	15.21 (12.50, 18.49)	3.9	5.2	12.2	34.5	111.0	0
Cadmium (μg/L)	0.45 (0.41, 0.50)	0.19	0.30	0.47	0.69	1.07	0
Antimony (μg/L)	0.05 (0.05, 0.06)	0.03	0.04	0.05	0.07	0.09	1
Lead (μg/L)	0.92 (0.82, 1.04)	0.44	0.69	1.00	1.48	2.02	0

Abbreviations used: *LOD* limit of detection, *p10* 10th percentile, *p25* 25th percentile, *p50* 50th percentile, *p75* 75th percentile, *SG* specific gravity

**Fig. 2:**
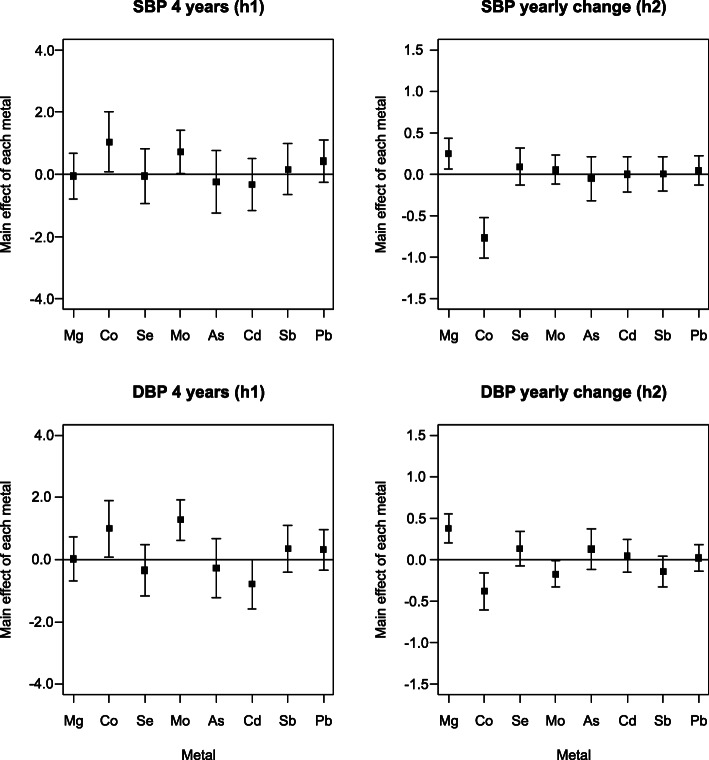
BVCKMR Main Effect Estimates for Each Mixture Component. Estimates for systolic blood pressure are shown on the top row, while estimates for diastolic blood pressure are shown on the bottom row. Plots in the left column show effect estimates for baseline blood pressure measures (age 4), while plots in the right column show effect estimates for the per-year change in blood pressure from age 4 to age 11. Effect estimates represent the difference in the outcome for an interquartile range increase in the specified metal, setting all other metals to their median value. Metals were log_2_-transformed, mean-centered, and scaled. Models were adjusted for maternal age, maternal education, maternal pre-pregnancy BMI, maternal smoking during pregnancy, child’s sex, child’s age, and child’s height. Abbreviations used: As, arsenic; Cd, cadmium; Co, cobalt; DBP, diastolic blood pressure; Mg, magnesium; Mo, molybdenum; Pb, lead; Sb, antimony; SBP, systolic blood pressure; Se, selenium

**Fig. 3:**
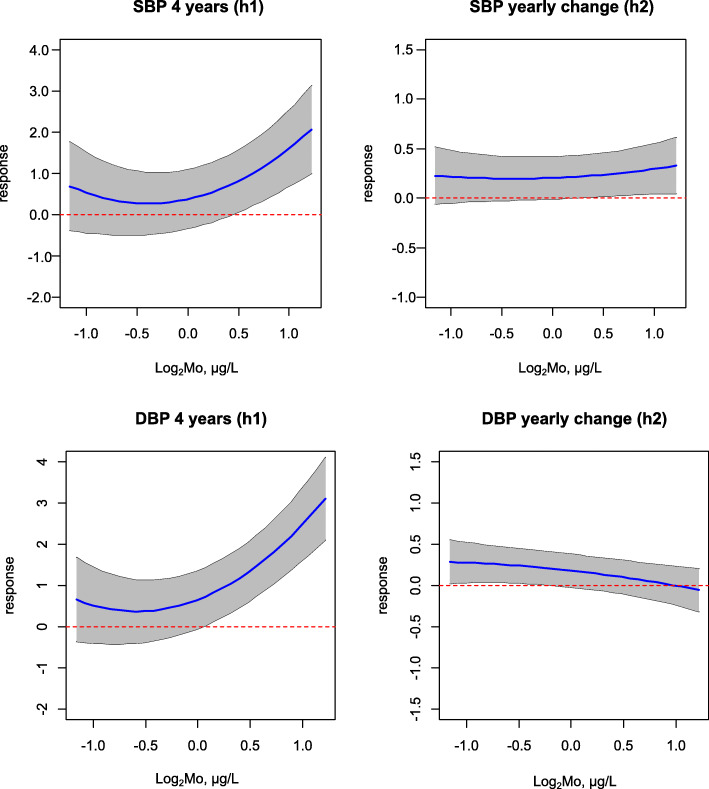
BVCKMR Estimated Exposure-Response Functions for Molybdenum. Exposure-response functions for systolic blood pressure are shown on the top row, while exposure-response functions for diastolic blood pressure are shown on the bottom row. Plots in the left column show exposure-response functions for baseline blood pressure measures (age 4), while exposure-response functions for per-year changes in blood pressure measures from age 4 to 11 are shown in the right column. Molybdenum was log_2_-transformed, mean-centered, and scaled. Models were adjusted for maternal age, maternal education, maternal pre-pregnancy BMI, maternal smoking during pregnancy, child’s sex, child’s age, and child’s height. Abbreviations used: DBP, diastolic blood pressure; Mo, molybdenum; SBP, systolic blood pressure

BVCKMR identified statistically significant associations between Mo and both SBP and DBP (evaluated continuously) at baseline (age 4) (Fig. 2). Setting other metals to their median, an interquartile range increase in Mo was associated with a 0.7 (95% CI: 0.0, 1.4) mmHg higher SBP and a 1.3 (95% CI: 0.6, 1.9) mmHg higher DBP at age 4 (Table S3). However, these relationships were found to be J-shaped, such that the positive associations between Mo and BP measures were driven by Mo concentrations above an inflection point of ~ 82 μg/L for SBP and ~ 52 μg/L for DBP (Fig. 3). In contrast, inverse associations were observed for Mo concentrations falling below this inflection point (Fig. 3). U- and J-shaped relationships were also observed between Mo and BP measures when using GAMMs (Fig. S3). In addition to these baseline associations, Mo was associated with significant longitudinal changes in DBP (Fig. 2, Table S3). BVCKMR estimated that an interquartile range increase in Mo was associated with a − 0.2 (95% CI: − 0.3, 0.0) mmHg lower per-year increase in DBP from ages 4 to 11 (setting other metals to their median), which was linear (Fig. 3, Table S3). Mo was not associated with significant changes in SBP over time (Fig. 3, Table S3).

**Fig. 4:**
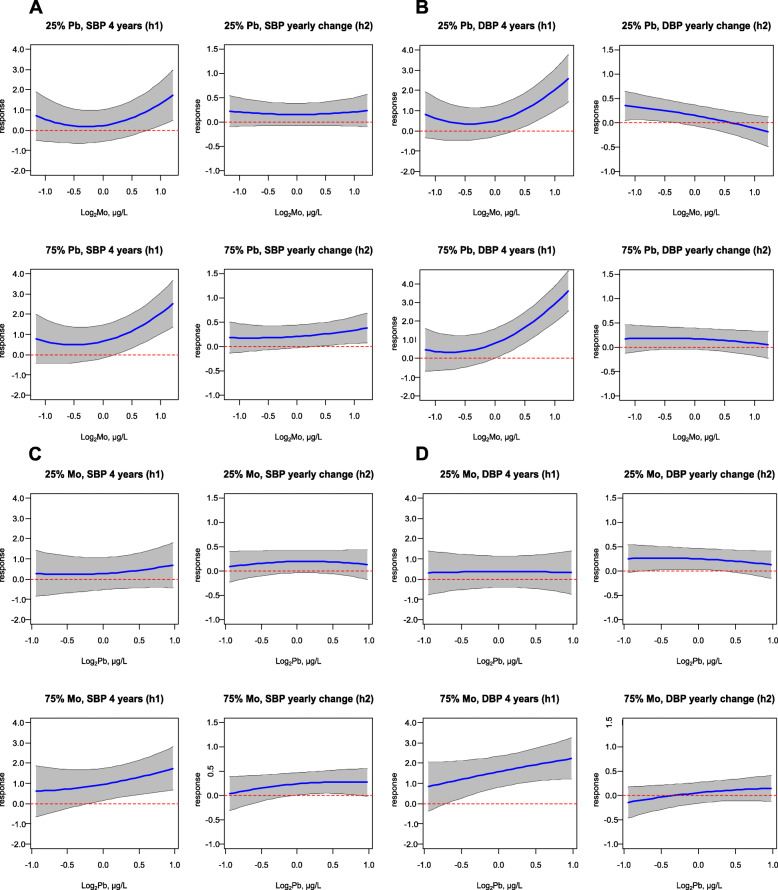
BVCKMR Estimated Bivariate Exposure-Response Functions for Molybdenum and Lead. For each panel, the top row shows the exposure-response function for metal 1 setting metal 2 to its 25th percentile, while the bottom row shows the exposure-response function for metal 1 setting metal 2 to its 75th percentile (with all other metals set to their median). The column on the left shows the exposure-response function for the baseline (age 4) blood pressure measure, while the column on the right shows the exposure-response function for the per-year change in the blood pressure measure. Panel (**a**) shows the molybdenum-systolic blood pressure relationship by lead level, panel (**b**) shows the molybdenum-diastolic blood pressure relationships by lead level, panel (**c**) shows the lead-systolic blood pressure relationship by molybdenum level, and panel (**d**) shows the lead-diastolic blood pressure relationship by molybdenum level. Molybdenum and lead were log_2_-transformed, mean-centered, and scaled. Models were adjusted for maternal age, maternal education, maternal pre-pregnancy BMI, maternal smoking during pregnancy, child’s sex, child’s age, and child’s height. Abbreviations used: DBP, diastolic blood pressure; Mo, molybdenum; Pb, lead; SBP, systolic blood pressure

Although overall associations between Pb and the continuous BP measures were null, both for BVCKMR and GAMMs (Fig. 2, Table S3, Fig. S3), significant pairwise interactions between Pb and Mo were identified by GAMMs (SBP: approximate p for joint smooth term < 0.01, DBP: approximate p for joint smooth term < 0.01) (Table S4). These interactions were confirmed visually by BVCKMR (Fig. 4). Positive associations were observed between Pb and continuous BP measures at age 4 when Mo levels were set to their 75th, but not 25th, percentile (with other metals set to their median) (Fig. 4). Associations between Mo and continuous BP measures at age 4 were also modified by Pb; for Mo concentrations above the inflection points, the positive associations between Mo and BP were stronger when Pb levels were set to their 75th, compared with 25th, percentile (setting other metals to their median) (Fig. 4).

Co was associated with significantly higher levels of both SBP and DBP (evaluated continuously) at baseline (Fig. 2). Setting other metals to their median, an interquartile range increase in Co was associated with a 1.0 (95% CI: 0.1, 2.0) mmHg higher SBP and a 1.0 (95% CI: 0.1, 1.9) mmHg higher DBP at age 4. However, these relationships were found to be non-linear, such that the positive associations were only observed for Co concentrations exceeding ~ 0.5 μg/L (Fig. S4). Co was also associated with significantly lower per-year increases in both SBP and DBP from ages 4 to 11 (Fig. 2, Table S3). Setting other metals to their median, BVCKMR estimated an interquartile range increase in Co to be associated with a − 0.8 (95% CI: − 1.0, − 0.5) mmHg lower per-year increase in SBP and a − 0.4 (95% CI: − 0.6, − 0.2) mmHg lower per-year increase in DBP during this period (Table S3). However, this association was non-linear for SBP, such that the lower per-year increase in SBP was only observed for Co concentrations below ~ 0.5 μg/L (Fig. S4). In contrast with the BVCKMR results, Co was not significantly associated with either SBP or DBP when evaluated individually using GAMMs (Fig. S3).

Mg was not significantly associated with continuous BP at baseline, but was associated with significantly higher per-year increases in both SBP and DBP from age 4 to 11 (Fig. 2, Table S3). Setting other metals to their median, BVCKMR estimated that an interquartile increase in Mg was associated with a 0.3 (95% CI: 0.1, 0.4) mmHg higher per-year increase in SBP and a 0.4 (95% CI: 0.2, 0.6) mmHg higher per-year increase in DBP. These longitudinal associations were driven by Mg concentrations > 66.9 mg/L (Fig. S5). Mg was not significantly associated with either SBP or DBP when evaluated individually using GAMMs (Fig. S3).

Although BVCKMR identified a significant inverse association between Cd and DBP, evaluated continuously at age 4, a similar association was not observed for SBP (Fig. 2, Table S3). Furthermore, Cd was not associated with longitudinal changes in either SBP or DBP (Fig. 2, Table S3), and GAMMs did not identify significant associations between Cd and either SBP or DBP (Fig. S3). Associations between remaining metals (As, Sb, Se) and continuous BP measures were consistently null across methods (Fig. 2, Table S3, Fig. S3).

**Fig. 5:**
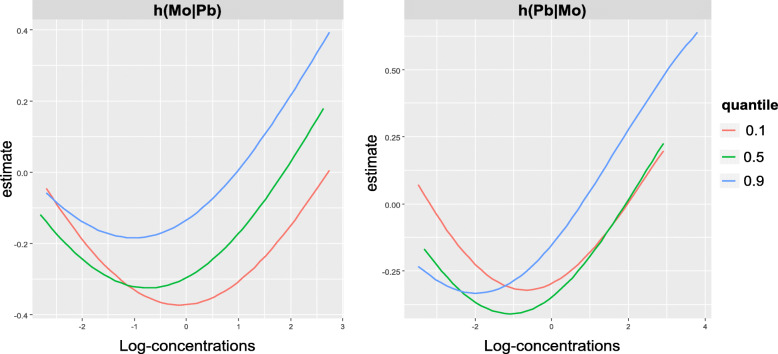
BKMR Bivariate Plots for Molybdenum and Lead with Elevated Blood Pressure at Age 11. The panel on the left shows the relationship between molybdenum and elevated blood pressure at age 11 for lead set to its 10th (red), 50th (green), and 90th (blue) percentile, with all other metals in the mixture set to their median. The panel on the right shows the relationship between lead and elevated blood pressure at age 11 for molybdenum set to its 10th (red), 50th (green), and 90th (blue) percentile, with all other metals in the mixture set to their median. Molybdenum and lead were log_2_-transformed, mean-centered, and scaled. Models were adjusted for maternal age, maternal education, maternal pre-pregnancy BMI, maternal smoking during pregnancy, child’s sex, child’s age, and child’s height. Abbreviations used: Mo, molybdenum; Pb, lead

The primary BKMR model for elevated BP at age 11 estimated similar group PIPs for toxic and essential elements (Table S5). Within the essential element group, Mo ranked highest, and within the toxic metal group, Pb ranked highest (Table S5). Similar to the BVCKMR results for Mo and continuous BP at age 4, BKMR identified a J-shaped relationship between Mo and elevated BP at age 11 (Fig. 5). A J-shaped relationship was also identified between Pb and elevated BP at age 11 (Fig. 5). Additionally, a possible interaction was identified between Mo and Pb for elevated BP at age 11, such that the inflection point for Mo decreased with increasing levels of Pb (~ 66 μg/L for Pb at its 10th percentile compared with ~ 46 μg/L for Pb at its median compared with ~ 42 μg/L for Pb at its 90th percentile) (Fig. 5). Similarly, the inflection point for Pb decreased with increasing levels of Mo (~ 0.6 μg/L for Mo at its 10th percentile compared with ~ 0.4 μg/L for Mo at its median compared with ~ 0.2 μg/L for Mo was at its 90th percentile) (Fig. 5). Other metals were not predictive of elevated BP at age 11 (Fig. S6). Similar to the BKMR results, when evaluating the metals individually using GAMs, U-shaped and J-shaped relationships were observed, respectively, for Mo and Pb in relation to elevated BP at age 11 (Fig. S7), although neither association was statistically significant (approximate *p* = 0.37 and 0.49, respectively). Furthermore, when using GAMs, inflection points of ~ 66 μg/L and ~ 0.4 μg/L were identified for Mo and Pb, respectively (Fig. S7), and a suggestive interaction was identified between Mo and Pb (approximate p for tensor product smooth term = 0.05) (Table S4).

BVCKMR results were similar after additionally adjusting for childhood ETS exposure at each time point, although the positive associations between Mo and baseline DBP and between Mg and the change in DBP were stronger (Table S6). The inverse association between Co and the change in DBP was also weaker after this adjustment, although still statistically significant (Table S6). BKMR results were also similar after additionally adjusting for ETS exposure at age 11 (Table S7, Fig. S8). Mo and Pb consistently ranked highest for their associations with elevated BP at age 11 (Table S7). J-shaped associations were observed between each metal and this outcome, consistent with the primary model (Fig. S8). When using the AAP percentile-based cutoffs for defining elevated BP at age 11, BKMR similarly ranked Mo and Pb highest for their associations with this outcome (Table S8, Fig. S9). However, bivariate relationships between Mo and Pb were inconsistent. In contrast with the primary model, the association between Mo and elevated BP at age 11 did not differ by Pb, and the U-shaped association between Pb and elevated BP at age 11 was only observed at low-to-moderate levels of Mo (Fig. S9).

## Discussion

In the current study, we examined the impact of prenatal metal mixture exposures on BP in a cohort of children in Greece, a country with a high prevalence of pediatric hypertension [32–34]. We used two mixture modeling approaches, including a novel method that can accommodate longitudinal data [37] and repeated BP measures spanning 7 years of follow-up to investigate the impact of prenatal exposure to a complex mixture of metals on BP trajectories in childhood and risk for elevated BP in early adolescence. Of the eight metals evaluated, Mo and Pb were most consistently associated with child BP. J-shaped associations were identified between each metal and continuous BP at age 4. Similar non-linear associations were also observed for each of these metals and elevated BP at age 11. Additionally, a possible synergistic interaction between Mo and Pb was identified for BP at age 4, which was robust across multiple methods.

There is sufficient evidence supporting a link between Pb exposure and increased risk for hypertension in adults [57], with possible mechanisms including alterations in the transport and distribution of calcium, decreased nitric oxide availability, dysregulation of vasoactive hormones, and increased oxidative stress and inflammation [58]. However, much less is known about the effects of Pb exposure on BP in early life. Although several studies have reported associations between prenatal Pb exposure and increased BP in children, these studies focused on a single time point in childhood [27, 29, 30]. In the current study, we examined the impact of prenatal Pb exposure as part of a larger mixture on BP at multiple time points in childhood (4, 6 and 11 years of age). Although our longitudinal mixture analysis did not find prenatal Pb to be associated with significant changes in BP across childhood, it was associated with higher BP measures at age 4 and was also predictive of elevated BP at age 11. These findings suggest that prenatal Pb does not accelerate age-associated increases in BP during childhood, but may increase BP in early childhood, contributing to elevated BP in adolescence. Interestingly, the associations between prenatal Pb exposure and BP at age 4 varied by Mo level. Positive associations were only observed at high concentrations of Mo, indicating a potential synergistic interaction between this pair of metals. A similar interaction was also identified between Pb and Mo in relation to elevated BP at age 11 in the primary analysis, which used static cutoffs to define this outcome [49], but this interaction was not robust when using the AAP percentile-based cutoffs [51]. We therefore cannot rule out the possibility that this interaction may be a chance finding. To our knowledge, interactions between Pb and Mo have not previously been examined in relation to BP.

Although less extensively studied compared with Pb, Mo has also been associated with increased BP and related cardiovascular outcomes [8, 59, 60]. Consistent with the current study, two studies in adults have similarly investigated and identified non-linear relationships between Mo and BP or other cardiovascular outcomes [59, 60]. One of these studies reported a U-shaped relationship between urinary Mo and hypertension with an inflection point (~ 60 μg/L) that falls within the range of inflection points identified for Mo and BP in our study of children (~ 42–82 μg/L) [59]. To our knowledge, previous studies have not investigated the longitudinal effects of Mo on BP in either children or adults. Although we found Mo to be associated with significantly smaller increases in DBP across childhood, the J-shaped relationship identified between Mo and BP at age 4 was similarly observed for elevated BP at age 11. Thus, the effects of Mo on BP in early childhood seem to persist into adolescence despite the longitudinal decreases observed for DBP. This is likely due to SBP remaining elevated from age 4 to 11. Interestingly, the associations between Mo and BP varied by Pb level. For example, the positive associations observed between high concentrations of Mo (> 82 μg/L for SBP and > 52 μg/L for DBP) and continuous BP at age 4 were stronger at high levels of Pb, which suggests that Pb may enhance the toxicity of Mo. While speculative, one potential mechanism by which Mo and Pb may jointly increase BP is through increased xanthine oxidase (XO) activity. XO is a Mo-dependent enzyme involved in purine metabolism which generates uric acid [61]. Previous studies have reported that Pb exposure may increase XO activity and uric acid levels [62–64], both of which have been associated with higher BP and increased risk for hypertension, including in children [65–69].

Although less consistent compared with findings for Pb and Mo, complex relationships were also identified between Co and BP, which differed by exposure level. For example, BVCKMR identified potential adverse effects of high concentrations of Co (> 0.5 μg/L) on continuous SBP and DBP at baseline, whereas low concentrations of Co (< 0.5 μg/L) were associated with protective effects longitudinally. However, Co was not predictive of elevated BP at age 11, and no clear patterns of association were observed between Co and any of the BP measures when this metal was evaluated individually using more traditional approaches (GAMMs and GAMs). Findings from previous studies on Co and BP have also been inconsistent. For example, while serum Co has been associated with reduced risk for pregnancy-induced hypertension and lower BP levels in children [13, 22], higher urinary Co concentrations have been reported among adults with elevated BP [8]. One potential explanation for these conflicting results may be the exposure levels represented by each population, as U-shaped relationships have previously been reported between Co and other outcomes, such as fetal growth [70], indicating protective effects at low (but not high) levels of exposure [41]. However, this would not explain the inconsistencies observed in the current study when using mixture modeling versus more traditional approaches. These differences are more likely explained by confounding from metal co-exposures, which is accounted for in mixture models (BVCKMR and BKMR) but not single metal analyses (GAMMs and GAMs).

Findings for other metals were unexpected or null. For example, when using BVCKMR we observed possible adverse effects of Mg (at high concentrations) on child BP (assessed continuously). This result is inconsistent with most [15, 17, 18], though not all [71], previous studies of Mg and BP. For example, one study of pregnant women found urinary Mg excretion in early pregnancy to be associated with increased SBP in late pregnancy [71]. Importantly, most studies investigating relationships between Mg and BP were conducted among non-pregnant adults, and we are unaware of any studies that have examined impacts of prenatal Mg on child BP. Another unexpected result was the inverse association observed between urinary Cd and DBP at age 4 when using BVCKMR. In contrast, previous studies in adults have largely observed adverse effects of Cd exposure on BP [10, 12], while null associations have generally been reported for children and adolescents [38, 72–75]. However, a recent cross-sectional analysis of children and adolescents in the United States similarly observed an inverse association between urinary Cd and BP [72], possibly due to unmeasured confounding from diet, which is the main source of Cd among non-smokers [40].

Despite prior evidence that As, Sb, and Se may impact BP [8, 10–12, 14–16, 28, 76–78], results for these metals were consistently null. For As and Sb, one potential explanation may be the low exposure levels in Rhea. A null association was also observed between prenatal As exposure and child BP in the New Hampshire Birth Cohort, which is similarly represented by As exposures in the low-to-moderate range [27], and associations between Sb and cardiovascular outcomes have only been observed in more highly exposed adults [8, 78–81]. While numerous studies have examined relationships between Se and BP, findings have been largely inconclusive, with protective, adverse, and null associations observed depending on the study population [14]. To our knowledge, only two studies have investigated these relationships in children [77, 82]. One study, which reported higher urinary Se concentrations, observed a positive and significant correlation with DBP [77]. However, the second study, which similarly evaluated Se in the context of a mixture but measured in blood, did not find Se to be predictive of child BP [82].

Identifying sources of Pb and Mo exposure for pregnant women in Greece is critical, given the consistent adverse associations observed with BP in early childhood in Rhea. Few studies have investigated metal exposures in Greek populations. However, food and drinking water are major sources of Pb for European populations [83], and elevated Pb in drinking water has been observed in certain regions of Greece, including Crete [84]. Contaminated drinking water may therefore be a possible source of Pb exposure for this population. Diet is likely the main source of exposure for Mo [44]. Although legumes are particularly rich sources of this element, major dietary sources can vary by population. In European adults, cereals and cereal-based products are important dietary sources of Mo [85]. In the U.S., yogurt consumption has also been identified as an important predictor of urinary Mo for pregnant women, while chili pepper consumption was the strongest dietary predictor of urinary Mo in a study of pregnant women in Mexico [86, 87]. Prenatal vitamin use has also been associated with urinary Mo in certain populations, but this likely reflects concurrent use of other supplements, as prenatal vitamins do not typically contain Mo [86, 88]. Another possible source of Mo exposure is pollution from coal combustion [44]. While coal is currently being phased out, it still contributes to a major fraction of electricity production in Greece [89].

The current study had many strengths, including the measurement and evaluation of multiple metals in early pregnancy and repeated child BP measurements across 7 years of follow up. We also used two novel mixture modeling approaches that can account for complex non-linear associations, as well as synergistic and antagonistic relationships. To our knowledge, this is the first study to use BVCKMR, a longitudinal mixture modeling approach, to investigate the impact of an environmental mixture on child BP trajectories. By applying this method, we were able to evaluate how exposure to a complex metal mixture during pregnancy influences longitudinal changes in BP across childhood, in addition to examining impacts on BP in both early childhood and adolescence.

Our study also had important limitations. Although the estimated prevalence of elevated BP at age 11 in Rhea was similar to previous reports of elevated BP among adolescents in Greece [32–34], it may be an overestimate, as it was based on a single BP measurement [51]. However, it is unlikely that measurement error would have differed by prenatal metals exposure. While urine is an accepted biomarker of exposure for As, Cd, Co, Mg, Mo, Sb, and Se, it is also important to acknowledge that blood is the preferred biomarker for Pb [46]. While urinary Pb does reflect some of the inter-individual differences in exposure, blood Pb is a more sensitive biomarker [46]. Our use of urinary Pb may have therefore biased results toward the null. The use of total urinary As as a biomarker of As exposure is also a limitation. While total urinary As can reflect inorganic As and its metabolites, which are toxic, it can also reflect non-toxic arsenicals derived from fish and seafood [90]. Results were similar after additionally adjusting models for maternal fish and seafood consumption, but we cannot rule out the possibility of residual confounding. Another important consideration is that urinary metals were measured at a single time point in early pregnancy (median gestational age at collection: 12 weeks). It is therefore possible that we did not capture the most sensitive exposure window for certain elements. Our use of a single spot urine sample also has limitations, as this may reflect only very recent exposure for some metals [91]. Finally, since BVCKMR requires a minimum of three measurements per participant, the current study was restricted to a relatively small number of participants (*N* = 176 for this study, compared with *N* = 1363 for the full Rhea cohort). Although urinary metals did not differ between participants and non-participants, the participants in the current analysis were older and more educated on average. There were also small but statistically significant differences in some of the child BP measures. We therefore cannot rule out the possibility of selection bias. Focusing on this restricted set of participants may have also limited statistical power, particularly for the analyses investigating elevated BP at age 11. Future studies which pool data across multiple cohorts may therefore be informative.

## Conclusions

Our findings suggest that high concentrations of Mo (> 40–80 μg/L) combined with Pb may increase BP in early childhood, contributing to elevated BP in adolescence. Although we also identified possible longitudinal effects of Co and Mg (in the context of a complex mixture) on child BP, results for these metals were null when using more traditional approaches and will require additional investigation. Our findings for Mo and Pb have important public health implications, as high BP in childhood and adolescence is predictive of hypertension and CVD in adulthood [2]. Identifying major sources of Mo and Pb exposures in this population is therefore essential.

## Supplementary Information

**Additional file 1.**

## Data Availability

The data that support the findings of this study may be provided by Dr. Leda Chatzi upon reasonable request.

## Abbreviations

As: Arsenic
BKMR: Bayesian Kernel Machine Regression
BP: Blood pressure
BVCKMR: Bayesian Varying Coefficient Kernel Machine Regression
Cd: Cadmium
Co: Cobalt
CVD: Cardiovascular disease
DBP: Diastolic blood pressure
ETS: Environmental tobacco smoke
GAMs: Generalized additive models
GAMMs: Generalized additive mixed models
LOD: Limit of detection
Mg: Magnesium
Mo: Molybdenum
Pb: Lead
PIP: Posterior inclusion probability
Sb: Antimony
SBP: Systolic blood pressure
Se: Selenium
