# Supplementary material for: Prenatal metal mixtures and child blood pressure in the Rhea mother-child cohort in Greece
Source: Environ Health. 2021 Jan 6;20:1. doi: 10.1186/s12940-020-00685-9 (PMC7789252; doi:10.1186/s12940-020-00685-9)
Supplement: Supplementary file 1 — Additional file 1. [file 12940_2020_685_MOESM1_ESM.docx]

**Supplemental Materials**


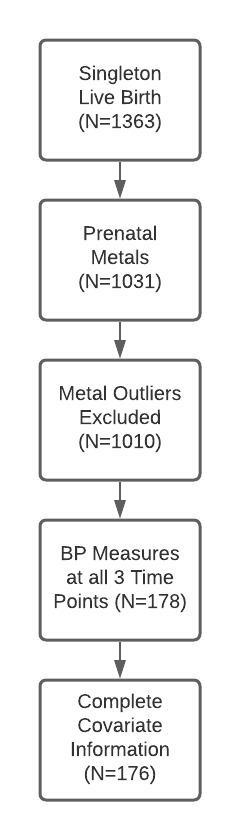


**Figure S1.** Flowchart of Participant Selection. Participants were excluded from the current study if they were missing maternal urinary metals data or were extreme outliers for any of the metals. Participants were also excluded if they were missing child BP measures for any of the three time points or covariate information. A total of 176 participants were eligible for the current study.

**Table S1.** Results for the Reference Materials Included in the ICP-MS Analyses for Quality Control

|  | **Seronorm™ Trace Elements Urine Blank, REF 201305, LOT OK4636** | | **Seronorm™ Trace Elements Urine, REF 201205, LOT NO2525** | |
| --- | --- | --- | --- | --- |
| Element | Reference value | Obtained value | Reference value | Obtained value |
| Magnesium (mg/L) | 89 ± 4 | 86 ± 4 | 54 ± 3.0 | 59 ± 3.2 |
| Cobalt (µg/L) | 0.28 ± 0.05 | 0.25 ± 0.01 | 10.1 ± 0.5 | 9.5 ± 0.46 |
| Selenium (µg/L) | 21.7 ± 2.8 | 25.1 ± 1.4 | 66.9 ± 7.1 | 75.7 ± 4.7 |
| Molybdenum (µg/L) | 61.4 ± 3.9 | 57.1 ± 2.8 | 42.3 ± 1.3 | 42.2 ± 2.1 |
| Arsenic (µg/L) | 85 ± 5 | 91 ± 4.7 | 184 ± 17 | 170 ± 8.2 |
| Cadmium (µg/L) | 0.31 ± 0.05 | 0.19 ± 0.01 | 5.06 ± 0.22 | 5.0 ± 0.16 |
| Antimony (µg/L) | 19.4 ± 0.9 | 18.0 ± 0.54 | 102 ± 3 | 107 ± 5 |
| Lead (µg/L) | 0.75 ± 0.05 | 0.79 ± 0.07 | 91.1 ± 7.0 | 84.0 ± 5.1 |

Abbreviations used: ICP-MS, inductively coupled plasma mass spectrometry

**Figure S2.** Directed Acyclic Graph Outlining Hypothesized Relationships Between Variables. Green variables represent the exposure or predictors of the exposure. Blue variables represent the outcome or predictors of the outcome. Pink variables represent potential confounders (causes of both exposure and outcome). Green arrows represent causal pathways from the exposure (metal mixture) to the outcome (child blood pressure). Pink arrows represent backdoor (biased) pathways from the exposure to the outcome. The directed acyclic graph was created using Dagitty (1). Abbreviations Used: BP, blood pressure; ETS, environmental tobacco smoke; SES, socioeconomic status.

**Table S2.** Comparison of Rhea Mother-Child Pairs Included versus Excluded from the Current Study

|  |  |  |  |
| --- | --- | --- | --- |
|  | **Non-Participants (n=1,187)** | **Participants**  **(n=176)** |  |
| **Maternal Characteristics** | **N (%) or Mean (SD)** | **N (%) or Mean (SD)** | **p-value** |
| Maternal age at sample collection (years) | 29.3 (5.2) | 30.3 (4.2) | 0.015 |
| Pre-pregnancy BMI (kg/m^2^) | 24.2 (5.0) | 24.0 (4.0) | 0.607 |
| Maternal education |  |  | <0.001 |
| Low | 260 (23.0) | 18 (10.2) |  |
| Medium | 569 (50.4) | 90 (51.1) |  |
| High | 301 (26.6) | 68 (38.6) |  |
| Smoking during pregnancy |  |  | 0.681 |
| Never | 848 (75.8) | 136 (77.3) |  |
| Ever | 270 (24.2) | 40 (22.7) |  |
| Fish consumption (n=150) | 1.1 (1.6) | 1.0 (0.6) | 0.718 |
| 0 times/week | 114 (12.3) | 11 (7.3) | 0.186 |
| 1 time/week | 529 (56.9) | 87 (58.0) |  |
| >1 times/week | 286 (30.8) | 52 (34.7) |  |
| Gestational week at urine collection, median (IQR) | 12.0 (11.0, 15.0) | 12.0 (11.0, 15.0) | 0.957 |
| **Child Characteristics** |  |  |  |
| Sex |  |  | 0.175 |
| Male | 596 (50.2) | 98 (55.7) |  |
| Female | 591 (49.8) | 78 (44.3) |  |
| Environmental tobacco smoke exposure at 4 years | 286 (46.6) | 74 (42.8) | 0.375 |
| Environmental tobacco smoke exposure at 6 years | 144 (36.5) | 64 (36.4) | 0.983 |
| Environmental tobacco smoke exposure at 11 years | 37 (24.3) | 45 (25.6) | 0.798 |
| Overweight at 4 years | 91 (14.4) | 25 (14.2) | 0.644 |
| Obese at 4 years | 49 (7.7) | 10 (5.7) |  |
| Overweight at 6 years | 82 (21.0) | 35 (19.9) | 0.150 |
| Obese at 6 years | 52 (13.3) | 14 (8.0) |  |
| Overweight at 11 years | 41 (27.3) | 58 (33.3) | 0.526 |
| Obese at 11 years | 17 (11.3) | 20 (11.4) |  |
| SBP at 4 years (mmHg) | 90.4 (7.9) | 90.6 (7.6) | 0.734 |
| DBP at 4 years (mmHg) | 53.6 (5.3) | 53.7 (5.0) | 0.748 |
| SBP at 6 years (mmHg) | 95.5 (9.4) | 94 (8.2) | 0.059 |
| DBP at 6 years (mmHg) | 55.2 (6.8) | 53.9 (6.4) | 0.028 |
| SBP at 11 years (mmHg) | 103.7 (8.6) | 105.8 (9.4) | 0.038 |
| DBP at 11 years (mmHg) | 60.0 (6.7) | 61.2 (6.9) | 0.102 |
| **SG-Adjusted Urinary Metal Concentrations** |  |  |  |
| Magnesium (mg/L) | 76.5 (40.9) | 77.7 (41.7) | 0.714 |
| Cobalt (μg/L) | 0.8 (0.8) | 0.8 (0.7) | 0.791 |
| Selenium (μg/L) | 23.0 (8.7) | 22.8 (7.3) | 0.834 |
| Molybdenum (μg/L) | 68.8 (31.5) | 71.8 (32.9) | 0.259 |
| Arsenic (μg/L) | 50.1 (232.7) | 49.5 (138.2) | 0.973 |
| Cadmium (μg/L) | 0.5 (0.4) | 0.6 (0.4) | 0.336 |
| Antimony (μg/L) | 0.1 (0.1) | 0.1 (0.0) | 0.072 |
| Lead (μg/L) | 1.3 (0.9) | 1.2 (1.4) | 0.544 |

^a^p-value is from t-test (continuous) or Chi-square test (categorical)

^b^n=150

Abbreviations Used: IQR, interquartile range; SG, specific gravity

**Table S3.** Individual Metal Effect Estimates from BVCKMR^a^

|  | Baseline SBP (Age 4) | Per-Year Change in SBP | Baseline DBP (Age 4) | Per-Year Change in DBP |
| --- | --- | --- | --- | --- |
|  | Beta (95% CI) | Beta (95 % CI) | Beta (95% CI) | Beta (95% CI) |
| Mg | -0.1 (-0.8, 0.7) | **0.3 (0.1, 0.4)** | 0.0 (-0.7, 0.7) | **0.4 (0.2, 0.6)** |
| Co | **1.0 (0.1, 2.0)** | **-0.8 (-1.0, -0.5)** | **1.0 (0.1, 1.9)** | **-0.4 (-0.6, -0.2)** |
| Se | -0.1 (-0.9, 0.8) | 0.1 (-0.1, 0.3) | -0.3 (-1.2, 0.5) | 0.1 (-0.1, 0.3) |
| Mo | **0.7 (0.0, 1.4)** | 0.1 (-0.1, 0.2) | **1.3 (0.6, 1.9)** | **-0.2 (-0.3, 0.0)** |
| As | -0.3 (-1.3, 0.8) | -0.1 (-0.3, 0.2) | -0.3 (-1.2, 0.7) | 0.1 (-0.1, 0.4) |
| Cd | -0.3 (-1.2, 0.5) | 0.0 (-0.2, 0.2) | **-0.8 (-1.6, 0.0)** | 0.1 (-0.2, 0.2) |
| Sb | 0.5 (-0.7, 1.0) | 0.0 (-0.2, 0.2) | 0.4 (-0.4, 1.1) | -0.1 (-0.3, 0.0) |
| Pb | 0.4 (-0.3, 1.1) | 0.1 (-0.1, 0.2) | 0.3 (-0.3, 1.0) | 0.0 (-0.1, 0.2) |

^a^Metals were log_2_-transformed, mean-centered, and scaled. Models were adjusted for maternal age, maternal education, maternal pre-pregnancy BMI, maternal smoking during pregnancy, child’s sex, child’s exact age, and child’s height at each time point. Statistically significant associations are bolded.

Abbreviations used: As, arsenic; Cd, cadmium; Co, cobalt; DBP, diastolic blood pressure; Mg, magnesium; Mo, molybdenum; Pb, lead; Sb, antimony; SBP, systolic blood pressure; Se, selenium.

**A**


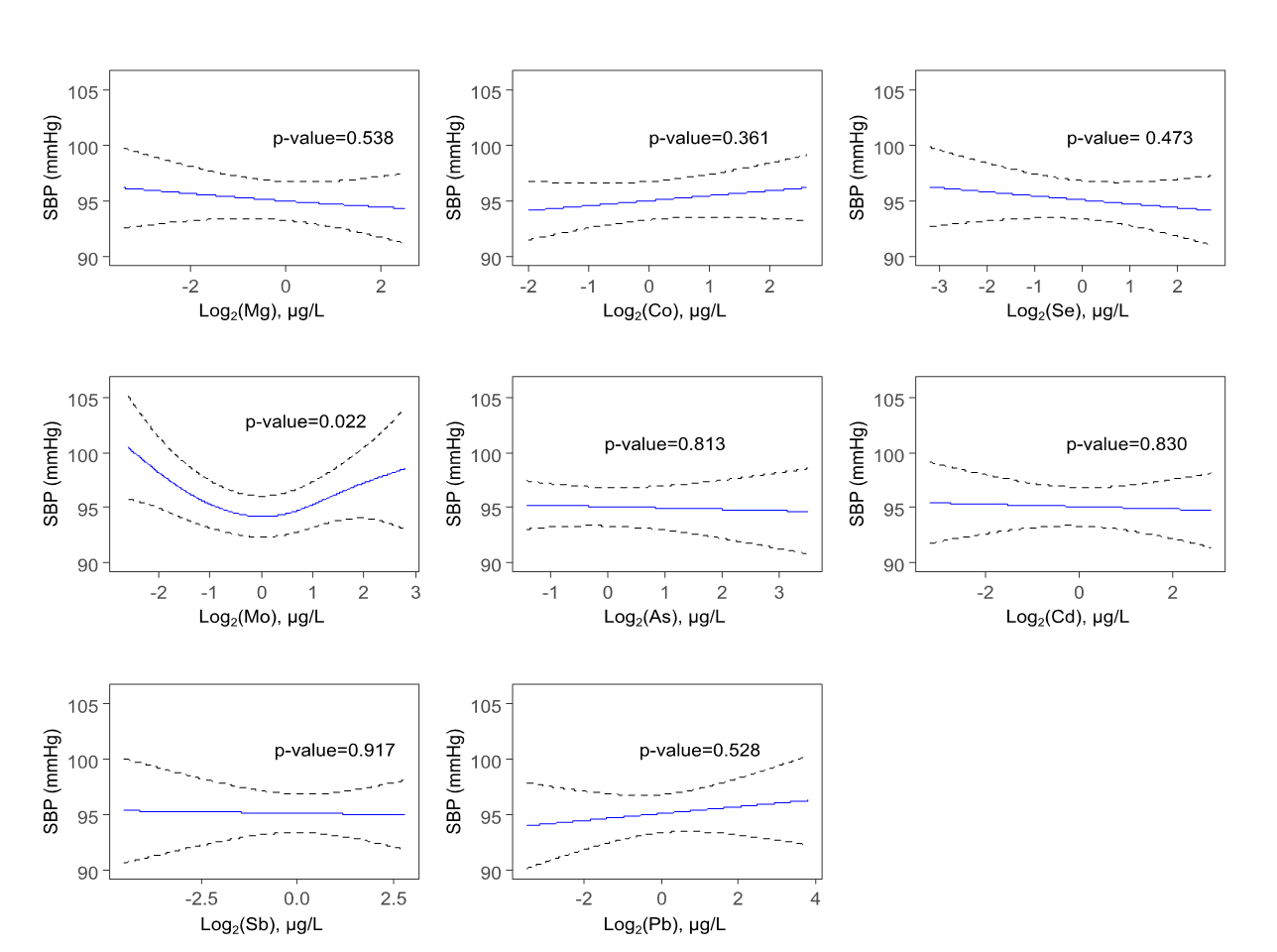


**B**


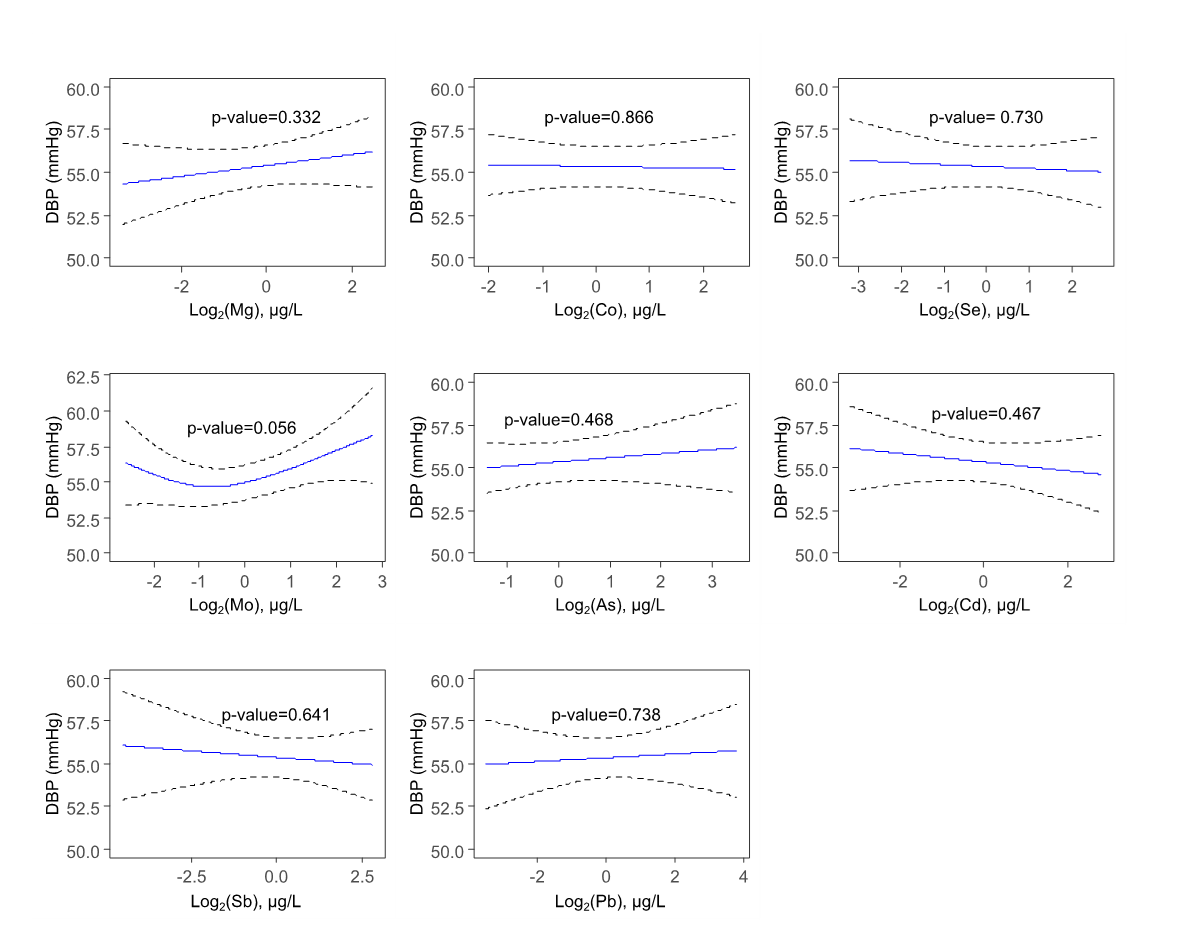


**Figure S3.** Generalized Additive Mixed Models. Metals were evaluated individually in relation to systolic blood pressure (**A**) and diastolic blood pressure (**B**), measured at 4, 6, and 11 years of age. Metals (log_2_-transformed, mean-centered, and scaled) are presented on the x-axis, and the predicted blood pressure measure is presented on the y-axis. Models were adjusted for maternal age, maternal education, maternal pre-pregnancy BMI, maternal smoking during pregnancy, child’s sex, child’s exact age, and child’s height at each time point.

Abbreviations used: As, arsenic; Cd, cadmium; Co, cobalt; DBP, diastolic blood pressure; Mg, magnesium; Mo, molybdenum; Pb, lead; Sb, antimony; SBP, systolic blood pressure; Se, selenium.

**Table S4.** Metal Pairwise Interaction P-Values Derived from Generalized Additive Mixed Models and Generalized Additive Models

| Metal Pair | SBP^a^ | DBP^a^ | Elevated BP  at Age 11^b^ |
| --- | --- | --- | --- |
| ti(Mg,Co) | 0.62 | 0.19 | 0.48 |
| ti(Mg,Se) | 0.11 | 0.58 | 0.42 |
| ti(Mg,Mo) | 0.28 | **0.01** | 0.51 |
| ti(Mg,As) | 0.90 | 0.60 | 0.72 |
| ti(Mg,Cd) | 0.64 | 0.06 | 0.39 |
| ti(Mg,Sb) | 0.11 | **0.02** | 0.89 |
| ti(Mg,Pb) | 0.11 | 0.16 | 0.54 |
| **ti(Co,Se)** | **0.05** | 0.53 | 0.55 |
| **ti(Co,Mo)** | **0.04** | 0.36 | 0.19 |
| **ti(Co,As)** | **<0.01** | **0.01** | 0.82 |
| ti(Co,Cd) | 0.69 | 0.87 | 0.69 |
| ti(Co,Sb) | 0.06 | 0.29 | 0.98 |
| ti(Co,Pb) | 0.48 | 0.20 | 0.09 |
| ti(Se,Mo) | 0.99 | 0.11 | 0.90 |
| ti(Se,As) | 0.75 | 0.69 | 0.27 |
| ti(Se,Cd) | 0.15 | 0.08 | 0.19 |
| ti(Se,Sb) | 0.05 | 0.07 | 0.62 |
| ti(Se,Pb) | 0.06 | 0.99 | 0.17 |
| ti(Mo,As) | 0.40 | 0.70 | 0.57 |
| ti(Mo,Cd) | 0.28 | 0.07 | 0.85 |
| ti(Mo,Sb) | 0.30 | 0.99 | 0.22 |
| **ti(Mo,Pb)** | **0.01** | **0.01** | **0.05** |
| ti(As,Cd) | 0.60 | 0.27 | 0.51 |
| ti(As,Sb) | 0.56 | 0.52 | 0.73 |
| **ti(As,Pb)** | **0.03** | 0.14 | 0.14 |
| **ti(Cd,Sb)** | **0.03** | 0.15 | 0.76 |
| ti(Cd,Pb) | 0.52 | 0.28 | 0.45 |
| ti(Sb,Pb) | 0.64 | 0.22 | >0.99 |

^a^Generalized additive mixed models were adjusted for maternal age, maternal education, maternal pre-pregnancy BMI, maternal smoking during pregnancy, child’s sex, child’s exact age, and child’s height at each time point. Metals were log_2_-transformed, mean-centered, and scaled. Statistically significant p-values (<0.05) are bolded.

^b^Generalized additive models were adjusted for maternal age, maternal education, maternal pre-pregnancy BMI, maternal smoking during pregnancy, child’s sex, child’s exact age at the 11-year time point, and child’s height at the 11-year time point. Metals were log_2_-transformed, mean-centered, and scaled. Statistically significant p-values (<0.05) are bolded.

Abbreviations used: As, arsenic; BP, blood pressure; Cd, cadmium; Co, cobalt; DBP, diastolic blood pressure; Mg, magnesium; Mo, molybdenum; Pb, lead; Sb, antimony; SBP, systolic blood pressure; Se, selenium.

**Figure S4.** BVCKMR Estimated Exposure-Response Functions for Cobalt. Exposure-response functions for systolic blood pressure are shown on the top row, while exposure-response functions for diastolic blood pressure are shown on the bottom row. Plots in the left column show exposure-response functions for baseline blood pressure measures (age 4), while plots in the right column show exposure-response functions for per-year changes in blood pressure measures from age 4 to 11. Cobalt was log_2_-transformed, mean-centered, and scaled. Models were adjusted for maternal age, maternal education, maternal pre-pregnancy BMI, maternal smoking during pregnancy, child’s sex, child’s exact age, and child’s height at each time point.

Abbreviations used: Co, cobalt; DBP, diastolic blood pressure; SBP, systolic blood pressure.

**Figure S5.** BVCKMR Estimated Exposure-Response Functions for Magnesium. Exposure-response functions for systolic blood pressure are shown on the top row, while exposure-response functions for diastolic blood pressure are shown on the bottom row. Plots in the left column show exposure-response functions for baseline blood pressure measures (age 4), while exposure-response functions for per-year changes in blood pressure measures from age 4 to 11 are shown in the right column. Magnesium was log_2_-transformed, mean-centered, and scaled. Models were adjusted for maternal age, maternal education, maternal pre-pregnancy BMI, maternal smoking during pregnancy, child’s sex, child’s exact age, and child’s height at each time point.

Abbreviations used: DBP, diastolic blood pressure; Mg, magnesium; SBP, systolic blood pressure.

**Table S5.** BKMR Posterior Inclusion Probabilities for Elevated BP at Age 11^a^

|  |  |  |  |
| --- | --- | --- | --- |
| Metal | Group | Group PIP | Conditional PIP |
| Mg | Essential | 0.69 | 0.18 |
| Co | Essential | 0.69 | 0.15 |
| Se | Essential | 0.69 | 0.21 |
| **Mo** | **Essential** | **0.69** | **0.46** |
| As | Toxic | 0.61 | 0.15 |
| Cd | Toxic | 0.61 | 0.15 |
| Sb | Toxic | 0.61 | 0.16 |
| **Pb** | **Toxic** | **0.61** | **0.55** |

^a^Group and conditional posterior inclusion probabilities are from Bayesian Kernel Machine Regression models for elevated blood pressure at age 11 in which metals were grouped into essential versus toxic elements. Models were adjusted for maternal age, maternal education, maternal pre-pregnancy BMI, maternal smoking during pregnancy, child’s sex, child’s exact age at the 11-year time point, and child’s height at the 11-year time point. Metals were log_2_-transformed, mean-centered, and scaled. Elevated BP at age 11 was defined according to the static cutoffs proposed by Xi et al. (2)

Abbreviations used: BKMR, Bayesian Kernel Machine Regression; PIP, posterior inclusion probability

**Figure S6.** BKMR Results for All Metals in Relation to Elevated BP at Age 11. Each panel shows the exposure-response function for the specified metal, setting all other metals in the mixture to their median. Metals were log_2_-transformed, mean-centered, and scaled. Models were adjusted for maternal age, maternal education, maternal pre-pregnancy BMI, maternal smoking during pregnancy, child’s sex, child’s exact age, and child’s height. Elevated BP was defined according to the static definitions proposed by Xi et al. (2).

Abbreviations used: As, arsenic; BKMR, Bayesian Kernel Machine Regression; BP, blood pressure; Cd, cadmium; Co, cobalt; Mg, magnesium; Mo, molybdenum; Pb, lead; Sb, antimony; Se, selenium


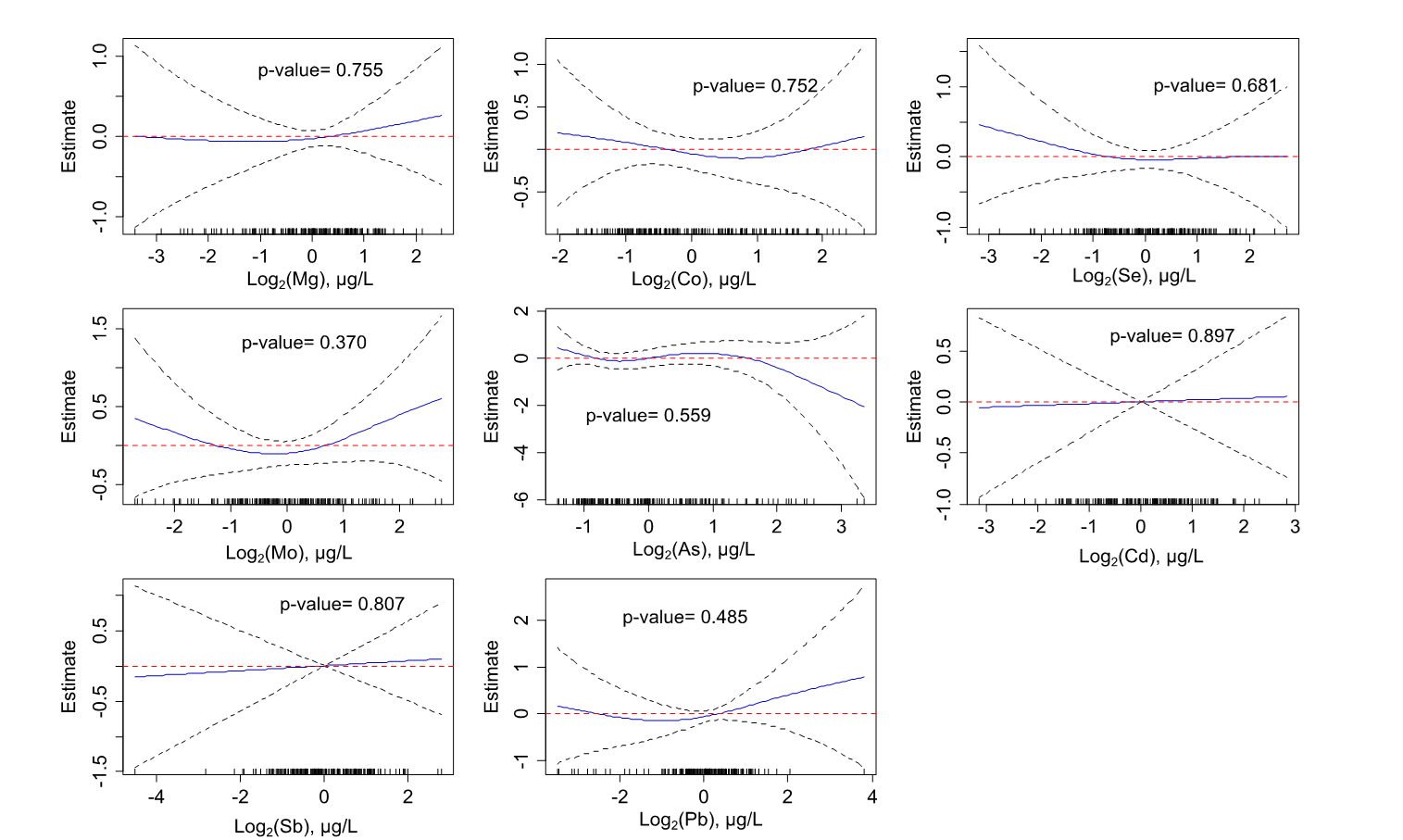


**Figure S7.** Generalized Additive Models for Elevated BP at Age 11. Metals were evaluated individually in relation to elevated blood pressure at age 11. Metals (log_2_-transformed, mean-centered, and scaled) are presented on the x-axis, and the predicted log-odds of elevated blood pressure at age 11 is on the y-axis. Models were adjusted for maternal age, maternal education, maternal pre-pregnancy BMI, maternal smoking during pregnancy, child’s sex, child’s exact age, and child’s height at each time point. Elevated BP was defined according to the static definitions proposed by Xi et al. (2)

Abbreviations used: As, arsenic; BP, blood pressure; Cd, cadmium; Co, cobalt; DBP, diastolic blood pressure; Mg, magnesium; Mo, molybdenum; Pb, lead; Sb, antimony; SBP, systolic blood pressure; Se, selenium.

**Table S6.** Comparison of BVCKMR Results After Additional Adjustment for Child ETS Exposure^a^

| Base Model (N=173)^b^ | Baseline SBP  (Age 4) | Per-Year  Change in SBP | Baseline DBP  (Age 4) | Per-Year  Change in DBP |
| --- | --- | --- | --- | --- |
| Mg | -0.1 (-0.9, 0.7) | 0.2 (0.0, 0.4) | -0.1 (-0.9, 0.7) | 0.2 (0.0, 0.4) |
| Co | 1.0 (0.0, 2.0) | -0.7 (-0.9, -0.4) | 1.0 (0.0, 2.0) | -0.7 (-0.9, -0.4) |
| Se | 0.0 (-0.9, 0.9) | 0.2 (-0.1, 0.4) | 0.0 (-0.9, 0.9) | 0.2 (-0.1, 0.4) |
| Mo | 0.7 (0.0, 1.4) | 0.1 (-0.1, 0.3) | 0.7 (0.0, 1.4) | 0.1 (-0.1, 0.3) |
| As | -0.2 (-1.2, 0.9) | -0.2 (-0.5, 0.0) | -0.2 (-1.2, 0.9) | -0.2 (-0.5, 0.0) |
| Cd | -0.3 (-1.1, 0.6) | -0.1 (-0.3, 0.2) | -0.3 (-1.1, 0.6) | -0.1 (-0.3, 0.2) |
| Sb | 0.1 (-0.7, 0.9) | 0.0 (-0.2, 0.2) | 0.1 (-0.8, 0.9) | 0.0 (-0.2, 0.2) |
| Pb | 0.4 (-0.3, 1.1) | 0.1 (-0.1, 0.3) | 0.4 (-0.3, 1.1) | 0.1 (-0.1, 0.3) |
| Additionally Adjusted for  ETS (N=173)^b^ |  |  |  |  |
| Mg | -0.1 (-0.8, 0.7) | 0.2 (0.0, 0.4) | 0.0 (-0.7, 0.7) | 0.3 (0.1, 0.5) |
| Co | 1.0 (0.0, 2.0) | -0.7 (-0.9, -0.4) | 0.9 (0.0, 1.9) | -0.3 (-0.5, -0.1) |
| Se | 0.0 (-0.9, 0.9) | 0.1 (-0.1, 0.4) | -0.2 (-1.0, 0.7) | 0.2 (-0.0, 0.4) |
| Mo | 0.7 (0.0, 1.4) | 0.1 (-0.1, 0.3) | 1.4 (0.7, 2.0) | -0.1 (-0.3, 0.0) |
| As | -0.1 (-1.2, 0.9) | -0.2 (-0.5, 0.0) | -0.2 (-1.1, 0.8) | -0.1 (-0.3, 0.2) |
| Cd | -0.3 (-1.1, 0.6) | -0.1 (-0.3, 0.2) | -0.7 (-1.5, 0.1) | 0.0 (-0.2, 0.2) |
| Sb | 0.1 (-0.8, 0.9) | 0.0 (-0.2, 0.2) | 0.2 (-0.6, 1.0) | -0.1 (-0.3, 0.1) |
| Pb | 0.4 (-0.3, 1.1) | 0.1 (-0.1, 0.3) | 0.4 (-0.2, 1.1) | 0.1 (-0.1, 0.3) |

^a^Effect estimates with 95% CIs that did not span the null are bolded

^b^Three participants were missing information on ETS exposure at age 4, so this sensitivity analysis was restricted to the 173 participants who were not missing this information to ensure that results could be directly compared for the same study subset.

Abbreviations Used: As, arsenic; BVCKMR, Bayesian Varying Coefficient Kernel Machine Regression; Cd, cadmium; Co, cobalt; DBP, diastolic blood pressure; ETS, environmental tobacco smoke exposure; Mg, magnesium; Mo, molybdenum; Pb, lead; Sb, antimony; SBP, systolic blood pressure; Se, selenium

**Table S7.** BKMR Posterior Inclusion Probabilities for Elevated BP at Age 11, With and Without Additional Adjustment for ETS Exposure at Age 11^a^

|  |  | Base Model | | Adjusting for ETS | |
| --- | --- | --- | --- | --- | --- |
| Metal | Group | Group  PIP | Conditional PIP | Group  PIP | Conditional PIP |
| Mg | Essential | 0.69 | 0.18 | 0.69 | 0.20 |
| Co | Essential | 0.69 | 0.15 | 0.69 | 0.16 |
| Se | Essential | 0.69 | 0.21 | 0.69 | 0.19 |
| **Mo** | **Essential** | 0.69 | **0.46** | 0.69 | **0.45** |
| As | Toxic | 0.61 | 0.15 | 0.61 | 0.15 |
| Cd | Toxic | 0.61 | 0.15 | 0.61 | 0.16 |
| Sb | Toxic | 0.61 | 0.16 | 0.61 | 0.18 |
| **Pb** | **Toxic** | 0.61 | **0.55** | 0.61 | **0.51** |

^a^Group and conditional posterior inclusion probabilities are from Bayesian Kernel Machine Regression models for elevated blood pressure at age 11 in which metals were grouped into essential versus toxic elements. Models were adjusted for maternal age, maternal education, maternal pre-pregnancy BMI, maternal smoking during pregnancy, child’s sex, child’s exact age at the 11-year time point, and child’s height at the 11-year time point. In a sensitivity analysis we also investigated results after additional adjustment for ETS exposure at age 11. Metals were log_2_-transformed, mean-centered, and scaled. Elevated BP at age 11 was defined according to the static cutoffs proposed by Xi et al. (2)

Abbreviations used: BKMR, Bayesian Kernel Machine Regression; ETS, environmental tobacco smoke; PIP, posterior inclusion probability

**Figure S8.** BKMR Univariate Plot for Associations Between Each Metal and Elevated BP at Age 11 After Additional Adjustment for ETS Exposure. Each panel shows the exposure-response function for the specified metal, setting all other metals in the mixture to their median. Metals were log_2_-transformed, mean-centered, and scaled. Models were adjusted for maternal age, maternal education, maternal pre-pregnancy BMI, maternal smoking during pregnancy, child’s sex, child’s exact age, and child’s height.

Abbreviations used: As, arsenic; BKMR, Bayesian Kernel Machine Regression; BP, blood pressure; Cd, cadmium; Co, cobalt; ETS, environmental tobacco smoke; Mg, magnesium; Mo, molybdenum; Pb, lead; Sb, antimony; Se, selenium

**Table S8.** PIPs for BKMR Using the AAP Percentile-Based BP Cutoffs for Elevated BP at Age 11^a^

|  |  | Static BP Cutoffs  (Primary Model) | | AAP Percentile  BP Cutoffs | |
| --- | --- | --- | --- | --- | --- |
| Metal | Group | Group  PIP | Conditional PIP | Group  PIP | Conditional PIP |
| Mg | Essential | 0.69 | 0.18 | 0.86 | 0.04 |
| Co | Essential | 0.69 | 0.15 | 0.86 | 0.07 |
| Se | Essential | 0.69 | 0.21 | 0.86 | 0.09 |
| **Mo** | **Essential** | 0.69 | **0.46** | 0.86 | **0.80** |
| As | Toxic | 0.61 | 0.15 | 0.67 | 0.38 |
| Cd | Toxic | 0.61 | 0.15 | 0.67 | 0.23 |
| Sb | Toxic | 0.61 | 0.16 | 0.67 | 0.15 |
| **Pb** | **Toxic** | 0.61 | **0.55** | 0.67 | **0.24** |

^a^Group and conditional posterior inclusion probabilities are from Bayesian Kernel Machine Regression models for elevated blood pressure at age 11 in which metals were grouped into essential versus toxic elements. Models were adjusted for maternal age, maternal education, maternal pre-pregnancy BMI, maternal smoking during pregnancy, child’s sex, child’s exact age at the 11-year time point, and child’s height at the 11-year time point. Metals were log_2_-transformed, mean-centered, and scaled. For the primary model, elevated BP at age 11 was defined according to the static cutoffs proposed by Xi et al. (2). In a sensitivity analyses, we compared results when using the AAP percentile cutoffs to define elevated BP at age 11 (3).

Abbreviations used: AAP, American Academy of Pediatrics; BKMR, Bayesian Kernel Machine Regression; ETS, environmental tobacco smoke; PIP, posterior inclusion probability

**Figure S9.** BKMR Bivariate Plots for Molybdenum and Lead in Relation to Elevated BP at Age 11 Using the AAP Percentile-Based BP Cutoffs. The left panel shows the exposure-response function for molybdenum, setting lead to its 10^th^ (red), 50^th^ (green), and 90^th^ (blue) percentile and holding all other metals in the mixture at the median. Metals were log_2_-transformed, mean-centered, and scaled. Models were adjusted for maternal age, maternal education, maternal pre-pregnancy BMI, and maternal smoking during pregnancy.

Abbreviations used: AAP, American Academy of Pediatrics; BP, blood pressure; Mo, molybdenum; Pb, lead

**References:**

1. Textor J, van der Zander B, Gilthorpe MS, Liśkiewicz M, Ellison GT. Robust causal inference using directed acyclic graphs: the R package ‘dagitty’. International journal of epidemiology. 2016;45(6):1887-94.

2. Xi B, Zhang T, Li S, Harville E, Bazzano L, He J, et al. Can pediatric hypertension criteria be simplified? A prediction analysis of subclinical cardiovascular outcomes from the Bogalusa Heart Study. Hypertension. 2017;69(4):691-6.

3. Flynn JT, Kaelber DC, Baker-Smith CM, Blowey D, Carroll AE, Daniels SR, et al. Clinical practice guideline for screening and management of high blood pressure in children and adolescents. Pediatrics. 2017;140(3).
